# Infection or infestation? Clarifying terminology for human and zoonotic food- and waterborne parasites

**DOI:** 10.1016/j.fawpar.2026.e00324

**Published:** 2026-03-10

**Authors:** Jean Dupouy-Camet

**Affiliations:** Paris Cité University & French Veterinary Academy, France 18 route des Brûleries, 89500 Armeau, France

**Keywords:** Infection, Infestation, Foodborne parasites, Helminths, Parasitological terminology

## Abstract

The terms infection and infestation are often used interchangeably in parasitology, yet the distinction between them is fundamental to accurate scientific communication. An analysis of recent literature, specifically within the journal *Food and Waterborne Parasitology*, reveals a predominant use of “infection,” even when describing helminths that do not multiply within the human host, a context where “infestation” would be scientifically more precise. This paper clarifies these definitions, highlights biological exceptions, and proposes a streamlined guideline for human or zoonotic food- and waterborne parasites: “If a parasite multiplies within the host, the process should be termed an infection; if no multiplication occurs, it should be considered an infestation.” Adopting this convention would enhance terminological consistency and stimulate necessary discussion among parasitologists, ultimately benefiting research, clinical practice, and public health communication.

## Highlights

• *Infection* and *infestation* are distinct terms; correct use improves parasitology clarity.
• *Infection* involves parasite multiplication within the host; *infestation* does not.
• Protozoa always cause *infections* due to obligatory multiplication.
• Helminths usually cause *infestations*, with notable species-specific exceptions.

## 1 Introduction

An analysis of article titles regarding food- and waterborne parasites reveals that the term “infection” is currently applied regardless of the parasite type or life cycle. For instance, the journal *Food and Waterborne Parasitology*, founded in 2015, publishes “high quality research articles… on parasites that are transmissible to humans from food or water.” While this journal has become an essential resource in human and veterinary parasitology, a review of its summaries indicates that “infection” is used almost exclusively to describe all modes of parasitic attack. This paper argues that for strictly human or zoonotic food- and waterborne parasites, a return to the distinction between infection and infestation is scientifically warranted and more appropriate.

## 2 Infection vs. infestation

In parasitology, the distinction between infection and infestation is well recognized, particularly in countries with Romance languages (Latin origins). Infection denotes the entry (penetration) and subsequent multiplication of a parasite within the host, a concept consistent with the terminology used for viruses, bacteria, and fungi. Conversely, infestation indicates the presence of a parasite, typically on the body surface or within body cavities (e.g., gastrointestinal or respiratory tracts), without simultaneous multiplication (Euzéby, 2008; Porta, 2016).

Clinically, an infection implies a disseminated or invasive process, whereas an infestation is often more focal. In the context of foodborne parasites, clearer terminology emerges when considering host–parasite dynamics:

• **Protozoa:** Species such as *Giardia intestinalis*, *Cryptosporidium parvum*, and *Toxoplasma gondii* multiply within host tissues or luminal cavities. These are properly regarded as agents of infection.
• **Helminths:** Ingestion of *Ascaris* eggs or fluke metacercariae leads to larval migration through tissues. While this migration causes symptoms suggestive of an infectious process, the parasite itself does not multiply. Helminths acquired via the ingestion of infective stages generally result in a fixed number of adult or larval forms corresponding to the number of ingested stages. Consequently, infestation is the more accurate descriptor.

However, tissue penetration alone does not constitute infection; multiplication is the defining criterion. Furthermore, several helminth species deviate from the standard pattern, involving endogenous multiplication or autoinfection. In these complex cases, the terminology must be specific:

• ***Enterobius vermicularis***: Produces embryonated eggs permitting self-reinfection.
• ***Taenia solium***: A human host harboring the adult worm (infestation) may ingest eggs from their own gut, causing cysticercosis (infection).
• ***Trichinella* spp.**: Larvae ingested in meat mature in the gut, but their progeny invades muscle tissue, qualifying the process as an infection.
• ***Echinococcus* spp.**: A single egg yields a cyst containing numerous protoscoleces, each capable of development. As multiplication occurs, this applies as infection.

**Fig. 1:**
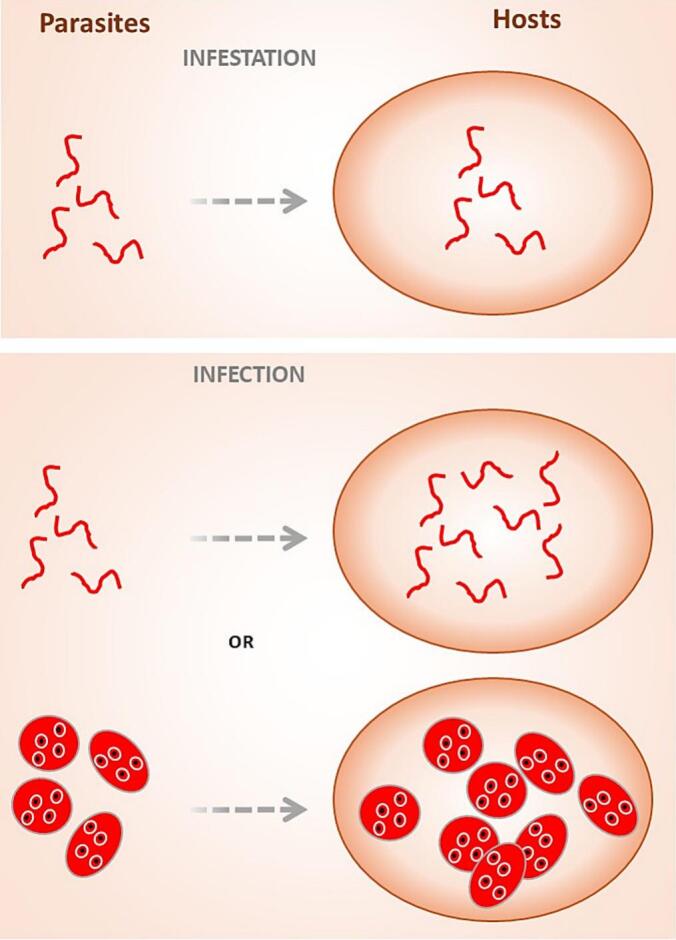
Schematic representation of *infection* versus *infestation* in food- and waterborne parasites.

These examples underscore that a single parasite species may involve both infestation and infection during different stages of its life cycle. The main food- and waterborne parasites are detailed in Table 1, alongside a proposal for the correct usage of infection versus infestation. (See Fig. 1.)

**Table 1:** Main food- and waterborne parasites and proposed use of the terms infection and infestation.

Parasite	Transmission stage	Tissue penetration	Multiplication in host	Outcome
*Entamoeba histolytica*	Cysts	Yes	Yes	Infection
*Giardia* sp.	Cysts	No	Yes	Infection
*Cryptosporidium* sp.	Oocysts	Yes	Yes	Infection
*Toxoplasma* sp.^1^	Oocysts / tissue cysts	Yes	Yes	Infection
Anisakids	Larvae	No	No	Infestation
*Ascaris* sp.	Eggs	Yes^2^	No	Infestation
*Trichuris trichiura*	Eggs	No	No	Infestation
*Enterobius vermicularis*	Eggs	No	Yes	Infection
*Trichinella* spp.	Larvae	Yes	Yes	Infection
*Taenia saginata*	Larvae	No	No	Infestation
*Taenia solium*	Larvae (eggs)	No (Yes^3^)	No (Yes)	Infestation (Infection)
*Dibothriocephalus* spp.	Larvae	No	No	Infestation
*Echinococcus* spp.	Eggs	Yes	Yes	Infection
Flukes^4^	Larvae	Yes^5^	No	Infestation

1. *Toxoplasma* sp.: Other modes of transmission include congenital transmission and organ transplantation.

2. Embryonated *Ascaris* eggs hatch in the intestine; larvae penetrate the intestinal mucosa, migrate via the bloodstream to the lungs, mature, penetrate the alveolar wall, ascend the bronchial tree, and are swallowed.

3. Humans infested with *Taenia solium* may undergo autoinfection from eggs from intestinal proglottids, leading to cysticercosis.

4. Includes *Fasciola* sp., *Clonorchis* sp., *Opistorchis* sp., *Fasciolopsi*s sp. and *Paragonimus* sp.

5. Metacercariae excyst in the duodenum, penetrate the intestinal wall into the peritoneal cavity, and migrate to target organs depending on species (lungs for *Paragonimus*, liver for *Fasciola*, *Clonorchis*, and *Opistorchis*).

## 3 Discussion

While the term “infection” is frequently used appropriately in *Food and Waterborne Parasitology*, it is often employed in contexts where “infestation” would be technically more precise, particularly for non-multiplying helminths. Although World Health Organization documents and standard textbooks frequently use the umbrella term of “parasitic infections” (WHO, 1987; Bruschi, 2014; Kaminsky and Mäser, 2025), earlier authors strictly preserved the distinction (Rogers, 1954), and it is occasionally discussed in depth (da Silva, 1997).

Notably, surgical (Hesse et al., 2012) and radiological (Abd El Bagi et al., 2004) literature often adopt “infestation” when referring to parasites in the gastrointestinal tract or biliary ducts. Regrettably, this distinction is largely neglected in general medicine. For example, the *Merck Manual of Diagnosis and Therapy* (Beers and Berkow, 1999) entitles its parasitology chapter “Parasitic Infections,” utilizing subchapters such as “Tapeworm infections.” However, the *Merck Manuals* (available online in multiple languages) reveal linguistic divergences (Merck Manuals, 2025). *Diphyllobothrium latum* is defined as “fish tapeworm infection” in English, but as “*infestation par le ténia du poisson*” in French. The German (*infektion*), Spanish (*infección*), and Italian (*infezione*) versions mirror the English usage. Interestingly, the *Merck Manuals* does use “infestation” regarding *Enterobius*, noting “enterobiasis is an infestation of the pinworm… and is the most common helminthic infection in the USA.”

These divergences support the need for precise definitions. Etymologically, “infection” derives from the Latin *infectio* (“to stain or corrupt”), a metaphor for an inner taint later applied to microbial invasion. “Infestation” derives from the Latin *infestatio* (“to attack or make unsafe”), originally describing an external assault, which narrowed to mean being overrun by parasites (Online Etymology Dictionary, 2025). Romance languages preserve this contrast, making the difference intuitive to speakers of French, Spanish, or Italian. In Germanic and other non-Latin languages, the distinction is less obvious, relying more on medical convention than etymology. This likely explains why the distinction remains robust among French-speaking parasitologists.

It is worth noting that Werner Apt (2013), in his textbook *Parasitologia Humana*, clearly differentiates the two terms. Furthermore, modern generative Artificial Intelligence tools often correctly distinguish between the two, based on biological criteria.

However, counter-arguments exist. Darwin Murrell, former president of the American Society of Parasitologists, notes that “infection” is so widely recognized by the public and scientific community that few are concerned with strict distinctions. He argues that science risks losing connection with the public, and that imposing a technical definition, one that narrows the common meaning of infection, might confuse stakeholders who support scientific research (personal communication, October 2025).

While this paper focuses on food- and waterborne parasites, the distinction is also relevant elsewhere. For vector-borne parasites, “infection” applies in most cases. For ectoparasites (ticks, lice, scabies), “infestation” is common, though debatable when multiplication occurs on the host (e.g., lice multiplying on hair vs. scabies mites multiplying within the skin). Nonetheless, given that *Food and Waterborne Parasitology* is a specialized scientific journal, terminological precision should be preserved within its pages.

## 4 Conclusion

To ensure scientific accuracy, the following operational guideline is suggested for human or zoonotic food- and waterborne parasites: “If a parasite multiplies within the host, the process should be termed an infection; if no multiplication occurs, regardless of development or metamorphosis, it should be considered an infestation.” Adoption of this convention would assist authors and editors in maintaining consistency and precision. It is hoped that this paper will stimulate further discussion within the readership of *Food and Waterborne Parasitology* and international parasitological associations, including the European and World Federations of Parasitologists, with the ultimate aim of promoting broader adoption of this terminology.

## Declaration of generative AI and AI-assisted technologies in the manuscript preparation process

During the preparation of this work the author used ChatGPT (OpenAI), Mistral AI Le Chat, and Gemini (Google) for assistance in refining terminology and improving the clarity of the English language in this manuscript. After using these tools, the author reviewed and edited the content as needed and takes full responsibility for the content of the published article.

## Credit authorship contribution statement

**Jean Dupouy-Camet:** Writing – original draft, Conceptualization.

## Declaration of competing interest

The authors declare that they have no known competing financial interests or personal relationships that could have appeared to influence the work reported in this paper.

Reports a relationship with that includes: has patent pending to. If there are other authors, they declare that they have no known competing financial interests or personal relationships that could have appeared to influence the work reported in this paper.
